# Functional ultrasound detects frequency-specific acute and delayed S-ketamine effects in the healthy mouse brain

**DOI:** 10.3389/fnins.2023.1177428

**Published:** 2023-05-17

**Authors:** Tudor M. Ionescu, Gillian Grohs-Metz, Bastian Hengerer

**Affiliations:** Boehringer Ingelheim, Ingelheim am Rhein, Germany

**Keywords:** functional ultrasound (fUS), S-ketamine, pharmacological imaging, frequency bands (range), functional connectivity

## Abstract

**Introduction:**

S-ketamine has received great interest due to both its antidepressant effects and its potential to induce psychosis when administered subchronically. However, no studies have investigated both its acute and delayed effects using *in vivo* small-animal imaging. Recently, functional ultrasound (fUS) has emerged as a powerful alternative to functional magnetic resonance imaging (fMRI), outperforming it in sensitivity and in spatiotemporal resolution. In this study, we employed fUS to thoroughly characterize acute and delayed S-ketamine effects on functional connectivity (FC) within the same cohort at slow frequency bands ranging from 0.01 to 1.25 Hz, previously reported to exhibit FC.

**Methods:**

We acquired fUS in a total of 16 healthy C57/Bl6 mice split in two cohorts (*n* = 8 received saline, *n* = 8 S-ketamine). One day after the first scans, performed at rest, the mice received the first dose of S-ketamine during the second measurement, followed by four further doses administered every 2 days. First, we assessed FC reproducibility and reliability at baseline in six frequency bands. Then, we investigated the acute and delayed effects at day 1 after the first dose and at day 9, 1 day after the last dose, for all bands, resulting in a total of four fUS measurements for every mouse.

**Results:**

We found reproducible (*r* > 0.9) and reliable (*r* > 0.9) group-average readouts in all frequency bands, only the 0.01–0.27 Hz band performing slightly worse. Acutely, S-ketamine induced strong FC increases in five of the six bands, peaking in the 0.073–0.2 Hz band. These increases comprised both cortical and subcortical brain areas, yet were of a transient nature, FC almost returning to baseline levels towards the end of the scan. Intriguingly, we observed robust corticostriatal FC decreases in the fastest band acquired (0.75 Hz–1.25  Hz). These changes persisted to a weaker extent after 1 day and at this timepoint they were accompanied by decreases in the other five bands as well. After 9 days, the decreases in the 0.75–1.25 Hz band were maintained, however no changes between cohorts could be detected in any other bands.

**Discussion:**

In summary, the study reports that acute and delayed ketamine effects in mice are not only dissimilar but have different directionalities in most frequency bands. The complementary readouts of the employed frequency bands recommend the use of fUS for frequency-specific investigation of pharmacological effects on FC.

## Introduction

S-ketamine, an N-methyl-D-aspartate (NMDA) receptor antagonist, has received tremendous attention in recent years due to its rapid antidepressant effects (Zarate et al., 2006; Schwartz et al., 2016), being the first non-monoaminergic compound approved by the food and drug administration for major depressive disorder treatment. Importantly, S-ketamine is a fast-acting antidepressant, exerting its effects rapidly and after just one dose, although the exact mechanisms of its antidepressant effects are still under debate (Kavalali and Monteggia, 2015; The Cochrane Collaboration et al., 2015). Additionally, within minutes after administration at subanesthetic doses, it has been shown to produce reliable dissociative effects (Krystal et al., 2005). Notably, an extensive body of literature has shown that blocking the NMDA receptors elicits transient symptoms and deficits similar to those observed in schizophrenia (Newcomer et al., 1999; Olney et al., 1999), in line with the glutamatergic hypothesis of schizophrenia (Olney and Farber, 1995). Therefore, it has also been extensively employed as a model of psychosis (Frohlich and Horn, 2014). Thus, understanding and quantifying the acute and delayed effects of S-ketamine is important both for the research of psychosis and for its clinical application as an antidepressant.

Blood-oxygenation-level-dependent functional magnetic resonance imaging (BOLD-fMRI) (Ogawa et al., 1990) studies are an important tool to generate *in vivo* biomarkers of brain function and dysfunction at rest and under activation (Gozzi and Zerbi, 2023). Specifically, many studies have used BOLD-fMRI to elucidate the effects of S-ketamine in both animals (Masaki et al., 2019) and humans (Mueller et al., 2018; Kotoula et al., 2021). More recently, functional ultrasound (fUS) has emerged as an alternative to probe neuronal activity *in vivo* via neurovascular coupling (Macé et al., 2011), providing higher temporospatial resolution and sensitivity compared to fMRI (Deffieux et al., 2018). Importantly, while both of hemodynamic nature, the Power Doppler signal recorded by fUS and the BOLD signal are not identical in nature (Deffieux et al., 2021) Specifically, the BOLD signal captures differences in magnetic susceptibility between oxygenated and deoxygenated hemoglobin and is therefore driven by changes in cerebral blood flow (CBF), cerebral blood volume (CBV) and cerebral metabolic rate of oxygen (CMRO_2_). However, to which extent each of these parameters contributes to the signal is still under debate (Buxton, 2012). The fUS signal is generated by the echoes produced by moving red blood cells acting as scatterers and has been shown to be proportional to the local hematocrit and, by extension, to the CBV (Macé et al., 2011; Mace et al., 2013; Montaldo et al., 2022). More recently, it has been demonstrated that fUS can also quantify changes in red blood cell velocity and cerebral blood flow in small vessels (Brunner et al., 2022). Therefore, it is purely hemodynamic in nature, and thus arguably more straightforward to interpret compared to the BOLD signal, which, as stated above, is also influenced by the CMRO_2_, in addition to the CBV and CBF.

Both methods can therefore be used to infer measures of functional connectivity (FC) (Biswal et al., 1995; Osmanski et al., 2014; Ferrier et al., 2020; Rabut et al., 2020; Vidal et al., 2020; De Paz and Macé, 2021; Vidal et al., 2022), derived from the temporal correlation between the timecourses of distinct brain areas. Since its emergence (Biswal et al., 1995), FC has been used to delineate the functional organizations of both human and animal brains, as well as changes occurring under different conditions such as diseases or pharmacological interventions, therefore emerging as an important *in vivo* biomarker for neuroscience (Khalili-Mahani et al., 2017; Spinosa et al., 2022). When measured through neurovascular coupling, FC has long been assumed to be restricted to low frequency ranges, typically between 0.01–0.1 Hz, due to the relatively slow, second-range canonical hemodynamic response function (Buxton, 2012). However, a recent body of literature has indicated that the hemodynamic response may be faster, and that functional connectivity measured using BOLD-fMRI may occur at many different frequency bands, from slow-5 (0.01–0.027 Hz) to slow-1 (0.5–0.75 Hz) (Boubela et al., 2013; Chen and Glover, 2015; Gohel and Biswal, 2015; DeRamus et al., 2021). Moreover, certain functional alterations may only occur at specific frequencies, underlining the importance of evaluating the connectome at different bands (Sasai et al., 2021). While not specifically developed for FC, the investigation of frequency-specific functional alterations has been shown to offer additional insight into pharmacological mechanisms, including those triggered by S-ketamine (Duan et al., 2022).

Here, we took advantage of the high temporal resolution of fUS, acquired in our study at 2.5 Hz, to study acute and delayed S-ketamine effects at distinct frequency bands investigated previously using BOLD-fMRI. First, we evaluated the reliability and reproducibility of the readouts derived at the specific frequencies at baseline and compared the global connectivity computed at each band with the results reported in the publication above as reference (Gohel and Biswal, 2015). Importantly, while the mentioned publication was confined to 0.5–0.75 Hz for the slow1 frequency band, using our data we could detect frequencies up to 1.25 Hz. Therefore, for comparison with the study above, we defined two bands for the slow-1 frequency, slow1-1 (0.5–0.75 Hz) and slow1-2 (0.75–1.25 Hz). After performing initial analyses to assess the robustness of the readouts at baseline (day-1) or under the application of saline, we examined the changes induced by S-ketamine directly after first application (day 0), as well as 24 h after first application (day 1) and after five repeated doses every 2 days (day 9). Our data pioneer the use of fUS for frequency-specific FC analysis and shed further light into the functional mechanisms of S-ketamine.

## Materials and methods

### Animals

This study was approved by and performed in accordance with the regulations of the respective authorities (Regierungspräsidium Tübingen, Germany and European Directive 2010/63/EU). The experiments were undertaken in C57BL/6 J male mice (age: 7–8 weeks; weight: 21–23 g) acquired from Charles River (Germany). The animals were kept in a 12:12 h light dark cycle.

The 16 animals were divided in two cohorts: 8 mice belonged to the S-ketamine cohort and 8 mice to the saline cohort. The animals were measured at day-1 (termed “baseline”), day 0 (termed “acute timepoint”), day 1 and day 9. A more detailed description of the experimental design can be found below. Some acquisitions had to be excluded due to heavy artifact pollution from an external source. Therefore, the final numbers used for the analysis were as follows: for the saline cohort 6 measurements at baseline, 6 acute measurements, 6 measurements at day 1 and 8 measurements at day 9. For the S-ketamine cohort, the following numbers of measurements were retained: 6 baseline measurements, 5 acute measurements, 6 measurements at day 1 and 8 measurements at day 9. Exemplary figures illustrating the pollution of the signal in a scan can be seen in the Supplementary material.

### Experimental design

**Figure 1:**
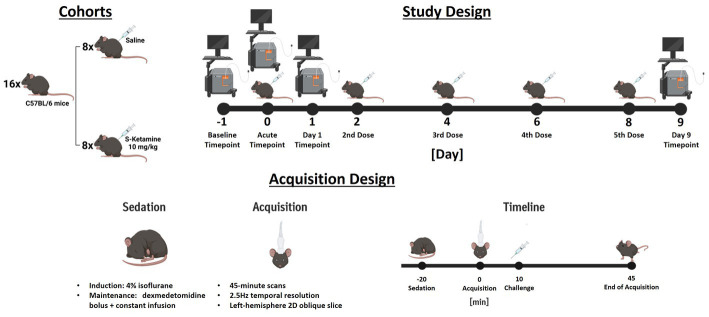
Summary of the experimental design. In study design, the injection days are depicted by an illustration of an injected mouse, while acquisition days are depicted by the illustration of an ultrasound scanner. For the challenge measurement, both illustrations indicate that the doses were administered during the acquisition. The measurement design summarizes the acquisition of challenge scans chronologically. For measurements different to the challenge timepoint no challenges were applied.

A summary of the experimental design is depicted in Figure 1.

Each animal received five doses of either 10 mg/kg S-ketamine (Sigma-Aldrich, St. Louis, MO) diluted at 2.5 mg/mL in saline (4 μL/g were injected), or corresponding volumes of saline solution. The doses were applied intraperitoneally during the acute measurement on day 0 (10 min into the acquisition), as well as at days 2, 4, 6, and 8. Therefore, the final measurements were performed 24 h after the fifth and final dose.

**Figure 2:**
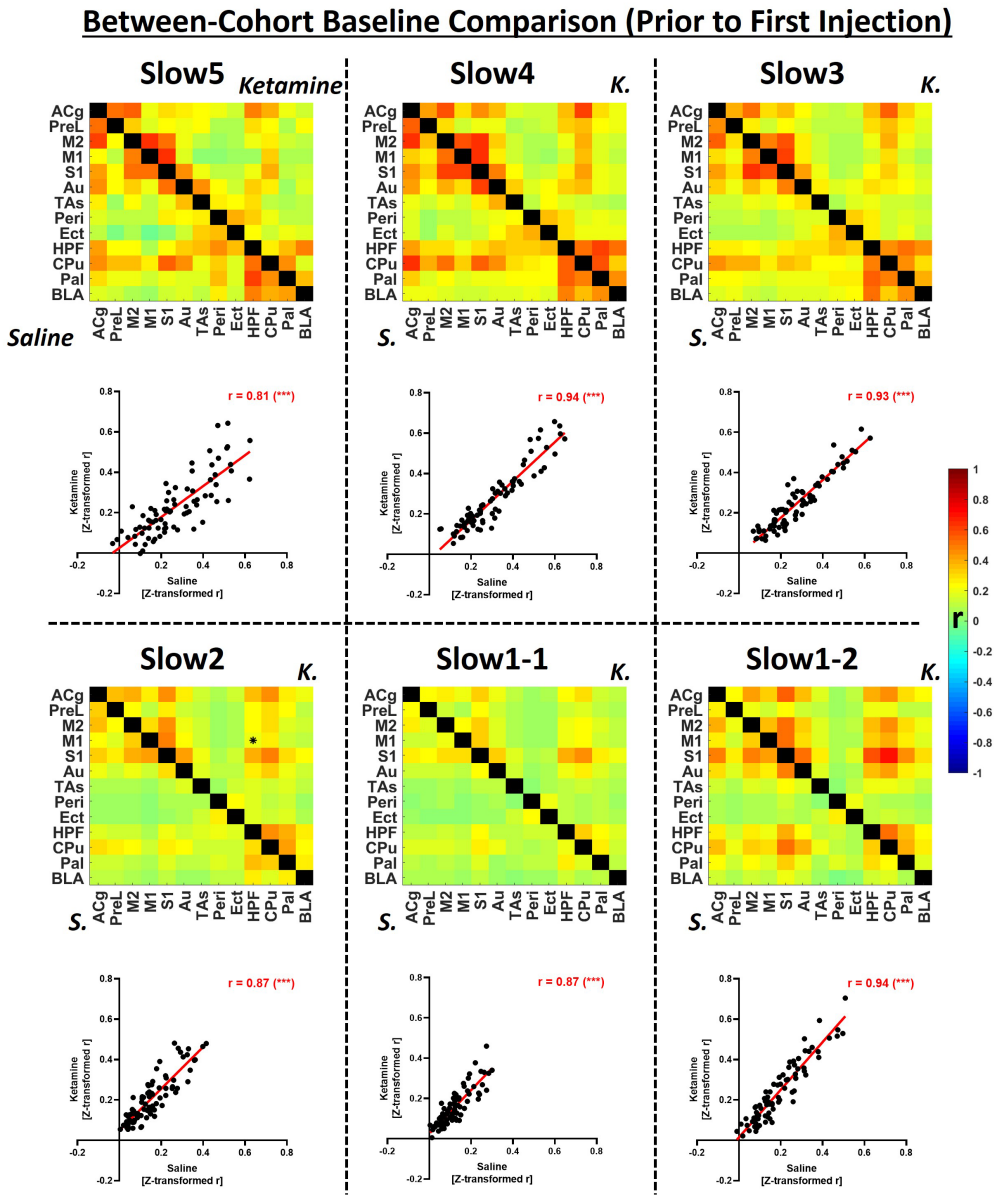
Group-level comparison of fUS-derived connectivity matrices between both cohorts within specific frequency bands at baseline. The matrices depict group-average connectivity at the baseline timepoint (Day-1) for the saline cohort (underneath diagonal) and for the ketamine cohort (above diagonal). The scatter plots indicate the correlations between the edge values computed for each cohort in the respective connectivity band. B, baseline; K, ketamine; ACg, anterior cingulate cortex; PreL, prelimbic cortex; M2, secondary motor cortex; M1, primary motor cortex; S1, primary sensory cortex; Au, auditory cortex; TAs, temporal association cortex; Ect, ectorhinal cortex; Peri, perirhinal cortex; HPF, hippocampal formation; CPu, caudoputamen; Pal, pallidum; BLA, basolateral amygdala, *r* = Fisher’s *z*-transformed Pearson’s *r* correlation coefficient. ***p < 0.001.

At the beginning of each imaging session, the sedation of the animals was induced using 4% isoflurane in oxygen. The isoflurane concentration was lowered and maintained at 1% for fixation in a stereotactic frame (David Kopf Instruments, United States) and insertion of the infusion catheter. Oxygen saturation and heart rate were monitored throughout the sessions and temperature maintained at 37.0°C using a PhysioSuite homeothermic warming system (Kent Scientific Corporation, United States). After stereotactic fixation, 0.05 mg/kg meloxicam (Metacam^®^, Boehringer Ingelheim, DE) was applied subcutaneously to minimize discomfort along with a bolus of 0.067 mg/kg dexmedetomidine hydrochloride (Tocris, United States). Sedation was then maintained by constant subcutaneous dexmedetomidine infusion (0.2 mg/kg/h, 5 mL/kg/h flow), while the isoflurane was gradually reduced by 0.1% every minute until reaching 0%. Directly after starting the dexmedetomidine constant infusion, the scalps of the mice were closely shaved, and ultrasonic gel was applied onto their scalps. Afterwards, the ultrasonic probe was lowered and placed ~1 mm above the scalp, ensuring complete immersion in the ultrasound gel. The probe included 128 piezoelectric transducers (0.08 mm per element) connected to a 128-channel scanner (Iconeus One, Iconeus, Paris, France). The images were generated using 200 compounded images acquired at a frame rate of 500 Hz. Each of these images was the sum of echoes at 11 different angles, all separated by 2° and ranging from −10° and 10° (Osmanski et al., 2014; Demené et al., 2015; Gesnik et al., 2017; Ferrier et al., 2020; Grohs-Metz et al., 2022). We first acquired an angiographic image generated from 30 coronal slices, each separated by 0.2 mm, ranging from the midbrain to the frontal cortex. We performed functional ultrasound acquisitions in a single oblique slice in the left hemisphere, shown in Supplementary Figure S3, the probe being positioned identically to our previous work (Grohs-Metz et al., 2022). The probe placement for the 2D functional ultrasound acquisitions was calculated using the Brain Positioning System (Nouhoum et al., 2021) (Iconeus, France = and encompassed several cortical and subcortical regions (please refer to Figure 2 for a list of all regions including abbreviations and to Supplementary Figure S3 for an illustration of the acquired ROIs overlaid over Power Doppler images from different sessions). The acquisitions were started 10 min after discontinuing isoflurane administration. Functional ultrasound measurements were acquired over 45 min, resulting in 6750 2D frames (0.4s/frame). After the imaging sessions, the mice received atipamezole (Alzane, Zoetis, Germany) to antagonize the medetomidine at a dose corresponding to five times the total applied dexmedetomidine.

All imaging experiments were performed using the small-animal Iconeus One fUS scanner (Iconeus, France). To emit ultrasonic plane waves and receive the backscattered Power Doppler signal a linear probe (15 MHz central frequency, Iconeus) was employed. A spatiotemporal singular value decomposition clutter filter extensively described previously (Demené et al., 2015) was applied to isolate blood signal and to generate one compiled image every 0.4 s (2.5 Hz frequency). For a detailed description of the image generation, please refer to (Macé et al., 2011).

### Data analysis

Regional Power Doppler time courses were extracted from 13 different brain areas in the left hemisphere. The raw Power Doppler signals were then scrubbed of artifacts using a protocol adapted from (Brunner et al., 2021). Next, the data were temporally filtered using a second-order Butterworth filter at the slow5 (0.01–0.027 Hz), slow4 (0.027–0.073 Hz), slow3 (0.073 Hz–0.198 Hz), slow2 (0.198–0.5 Hz), slow1-1 (0.5–0.75 Hz) and slow1-2 (0.75–1.25 Hz) frequency bands (please refer to Table 1 for reference).

**Table 1:** List of used frequency bands.

Frequency band name	Range
Slow5 Penttonen and Buzsáki (2003)	0.01–0.027 Hz Gohel and Biswal (2015) and Penttonen and Buzsáki (2003)
Slow4 Penttonen and Buzsáki (2003)	0.027–0.073 Hz Gohel and Biswal (2015) and Penttonen and Buzsáki (2003)
Slow3 Penttonen and Buzsáki (2003)	0.073–0.198 Hz Gohel and Biswal (2015) and Penttonen and Buzsáki (2003)
Slow2 Penttonen and Buzsáki (2003)	0.198–0.5 Hz Gohel and Biswal (2015) and Penttonen and Buzsáki (2003)
“Slow1-1” [Slow1 Penttonen and Buzsáki (2003) covered by Gohel and Biswal (2015)]	0.5–0.75 Hz Gohel and Biswal (2015) and Penttonen and Buzsáki (2003)
“Slow1-2” [Slow1 Penttonen and Buzsáki (2003) not covered by Gohel and Biswal (2015)]	0.75–1.25 Hz Buzsaki and Draguhn (2004) and Penttonen and Buzsáki (2003)

Using the filtered bands, functional connectivity matrices were generated for each animal and band by computing correlations between all pairs of regional time courses. The generated Pearson’s *r* correlation coefficients were then transformed to *Z*-scores using Fisher’s *Z*-transformation and averaged to derive group-level connectivity matrices. Regional and global connectivity strengths were computed by averaging either the correlations of one region to all other regions, or all the correlations in a matrix, respectively.

For static functional connectivity, the first 5 minutes of all acquisitions were excluded to ensure the stability of the signals and one matrix was generated for each animal and treatment group between 5 and 45 min after the start of the measurements. To assess reproducibility, we correlated all single edges (the correlation coefficient for an ROI–ROI pair) between the baseline measurements of the saline and ketamine cohorts at the group-level for all frequency bands. Furthermore, we derived the averaged global connectivity strengths for both cohorts and all bands at baseline to compare their distributions with the publication used as reference for such studies using BOLD-fMRI (Gohel and Biswal, 2015). To assess test–retest reliability and potential effects of the saline challenge, we employed the same analytical tools to compare the readouts of the saline cohort at baseline, at the acute timepoint, at day 1 and at day 9. To assess the effects of S-ketamine, we performed two-sample *t*-tests between S-ketamine and saline for all edges, as well as all regional and global connectivity strengths generated at the acute, day 1 and day 9 timepoints.

To achieve a more accurate temporal depiction of the acute effect of S-ketamine over the 35 min after the challenge was applied, we analyzed the FC dynamically using a sliding-window approach. Specifically, we used 5 min windows to generate correlation matrices, the end of the first window coinciding with the timepoint the challenges were applied. Then, we used 30 s sliding steps to compare the temporal development of FC directly after the challenge between both cohorts, testing for significance using two-sample *t*-tests. Edges significant after FDR correction, performed as described by Benjamini–Hochberg (Benjamini and Hochberg, 1995), as well as edges at *p* < 0.01 before correction are depicted in correlation matrices. For the acute acquisitions, the correction was performed over time, all other corrections were performed over edges.

## Results

### Connectivity readouts are repeatable and reliable at all frequency bands

In Figure 2, group-level baseline connectivity matrices (Day-1) of the saline and ketamine cohorts are presented, and the respective readouts are compared to assess reproducibility within individual frequency bands.

We detected only one significantly different edge (*p* < 0.01) between the baseline connectivities of the two cohorts at any frequency. Globally, we found the strongest connectivities at the slow5 (saline: 0.27 ± 0.05, ketamine: 0.23 ± 0.04), slow 4 (saline: 0.30 ± 0.07; ketamine: 0.27 ± 0.05), slow3 (saline: 0.27 ± 0.06; ketamine: 0.24 ± 0.06) and at slow1-2 (saline: 0.24 ± 0.13; ketamine: 0.27 ± 0.05), while the connectivity of both cohorts at slow2 (saline: 0.16 ± 0.04; ketamine: 0.21 ± 0.10), and slow 1-1 (saline: 0.12 ± 0.05; ketamine: 0.16 ± 0.10) were comparatively weaker. The connectivity patterns were consistent between the two cohorts, as assessed by correlating individual edges at each frequency band (Figure 2, scatter plots). Although all correlations were higher than 0.8, indicating very good repeatability, we found the highest values at the slow4, slow3 and, at slow1-2 (*r* = 0.94 for each). The correlations computed for slow5 (*r* = 0.81), slow2 (*r* = 0.87) and slow1-2 (*r* = 0.87) were slightly weaker.

**Figure 3:**
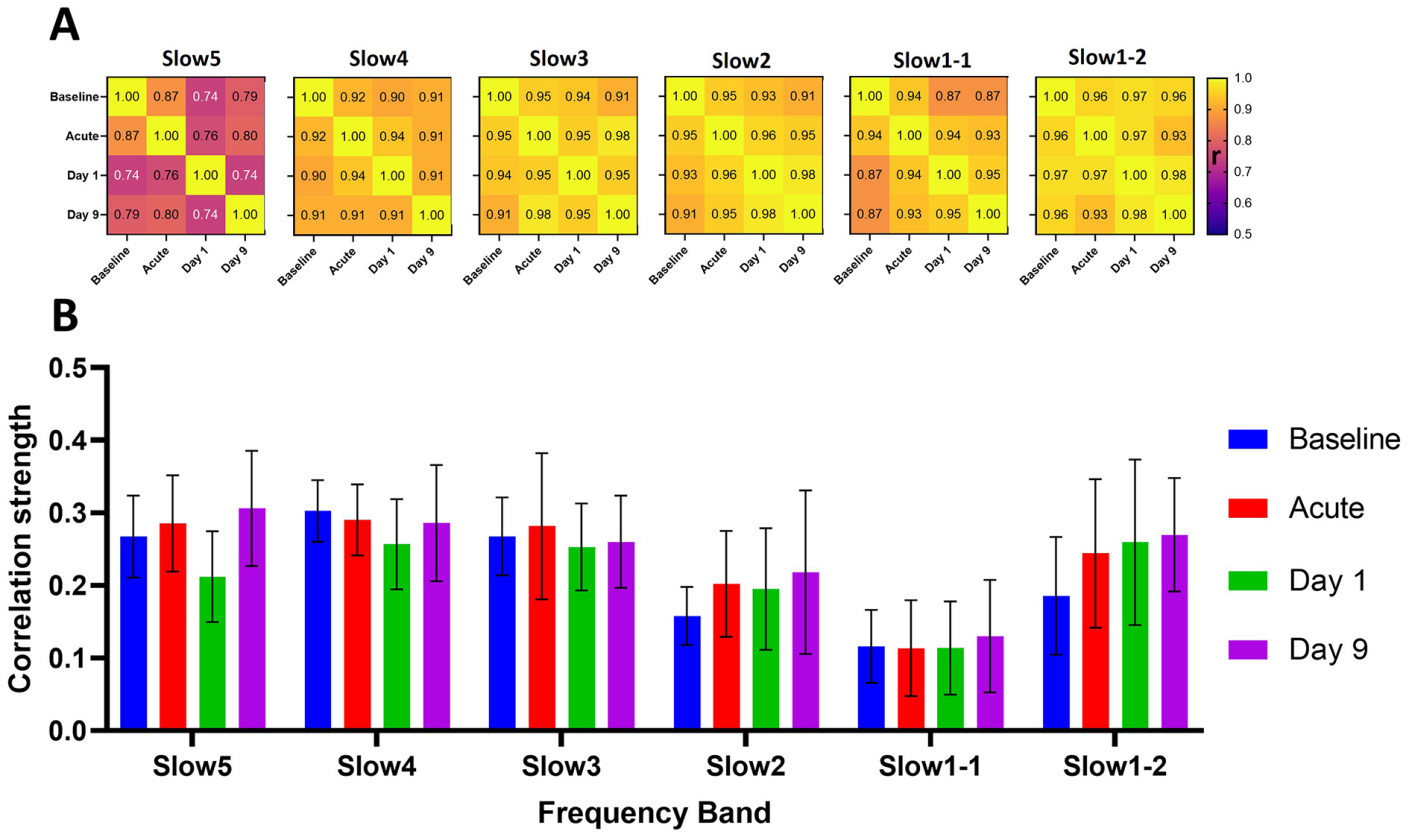
Longitudinal reliability and global connectivity of the saline cohort at the different timepoints and frequency bands. **(A)** The matrices show the correlations of the group-average connectivity patterns for each frequency band of the saline cohort. We compared the readouts of the four measurements performed by correlating the respective group-average edges generated at each timepoint in a pair-wise fashion to assess overall reliability of the computed group-average readouts. **(B)** The graph indicates the respective global connectivity strengths computed as the average of all edges contained in the corresponding connectivity matrices. The data are presented as mean ± SD. *r* = Pearson’s *r* correlation coefficient.

Figure 3 provides a longitudinal assessment of the test–retest reliability of the group-average readouts obtained from the saline cohort. First, we calculated correlation matrices, similar to those presented for the repeatability assessment in Figure 2. Then, we computed pair-wise correlations between all four timepoints (Figure 3, matrices). Furthermore, we assessed the distributions of global connectivity within the different frequency bands for every timepoint.

We found mostly consistent patterns of connectivity at all frequency bands when comparing the different timepoints (Figure 3A). For the slow4 (correlations from 0.90 and 0.94), slow3 (*r* = 0.91–0.98), slow2 (*r* = 0.96–0.98) and slow1-2 (*r* = 0.93–0.98) bands the correlations between the group-average readouts were higher than *r* = 0.9 for all pairwise comparisons between the different timepoints. For slow1-1 (*r* = 0.87–0.95) and particularly for the slow5 band (*r* = 0.74–0.87) we found slightly less consistent connectivity readouts. Furthermore, we observed comparable distributions of the global connectivity strengths of the different frequency bands at all timepoints (Figure 3B). Specifically, we found the highest global connectivities at the slow5 (*r* = 0.27 ± 0.04), slow4 (0.28 ± 0.02) and slow3 (*r* = 0.27 ± 0.01) bands. The global connectivity was at a lower level at all timepoints in the slow2 (*r* = 0.20 ± 0.03) and slow1-1 (0.12 ± 0.01) bands and again, interestingly, at a level similar to slow5-slow3 in the slow1-2 (0.24 ± 0.04) band. In summary, a consistent frequency distribution pattern emerged: slow5 ≈ slow4 ≈ slow3 > slow1-2 > slow2 > slow1-1 throughout all time points in the saline group, consistent with that observed in both groups at baseline.

### Acute effects of ketamine are frequency-specific

**Figure 4:**
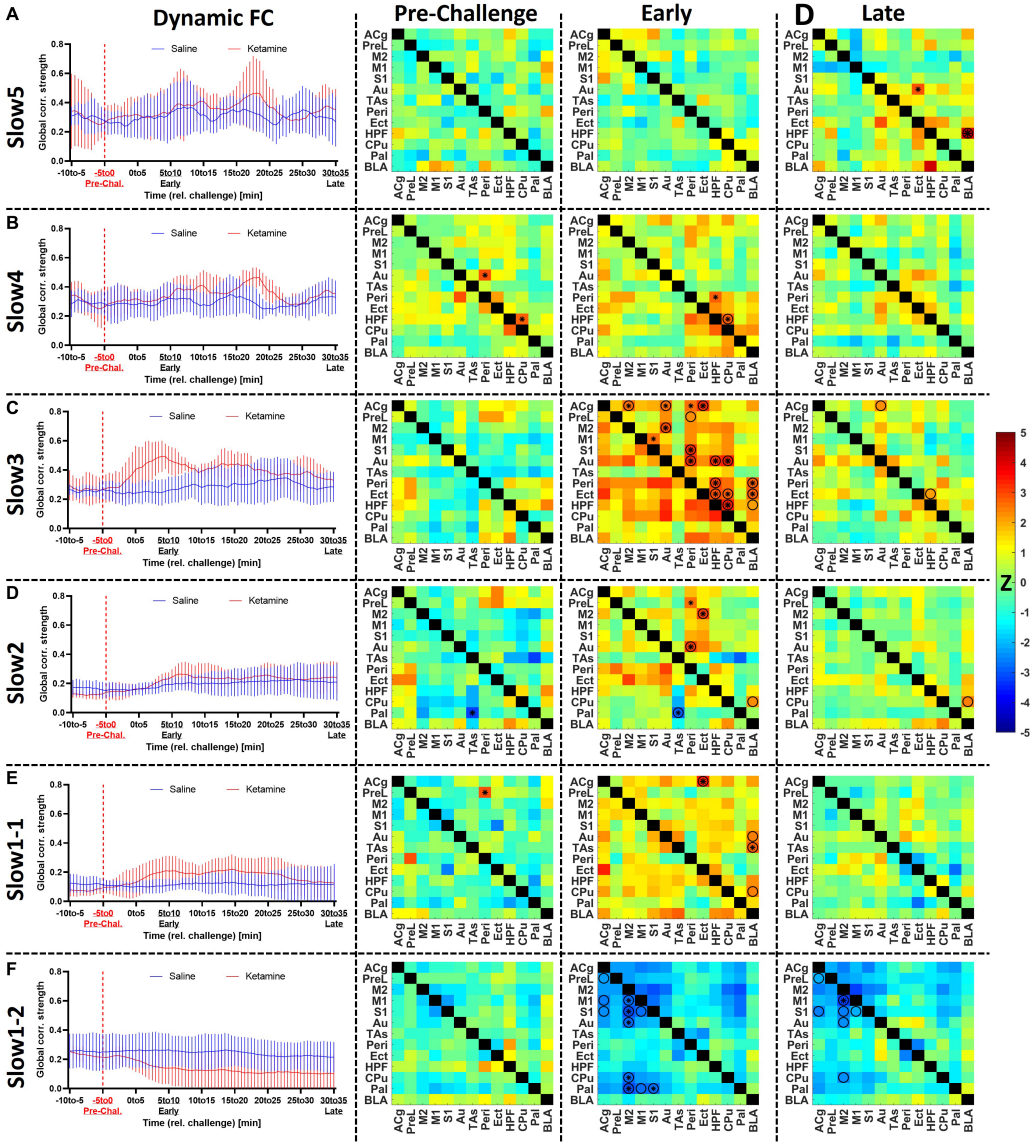
Assessment of the acute effects of ketamine on functional connectivity at **(A)** slow5, **(B)** slow4, **(C)** slow3, **(D)** slow2, **(E)** slow1-1, and **(F)** slow1-2. Left: dynamic global connectivity computed using a 5 min sliding window moved in 30 s steps for the two cohorts at all frequency bands. Right: the edge-level differences between cohorts are shown as *Z*-score matrices generated using two-sample *t*-tests between the two cohorts at baseline (−5 to 0 min relative to challenge), early (5–10 min) after the challenge and late (30–35 min) after the challenge. Negative *Z*-scores shown in cold colors indicate decreased connectivity induced by ketamine, while positive *Z*-scores represent enhanced FC connectivity compared to saline. * = *p* < 0.01 pre-FDR correction, *Z*-score > 2.58, *O* = *p* < 0.05 (FDR-corrected), combined * and *O* = *p* < 0.01 before FDR correction, *p* < 0.05 after FDR correction. *Z*, *Z*-score, for the abbreviations of all regions, please refer to Figure 2. Data presented as mean ± SD.

In Figure 4 we assessed the acute effects of ketamine on global FC strengths, as well as on edge level. The matrices depicted in this figure do not show correlation coefficients for both cohorts separately, but statistical differences expressed as *Z*-scores between both cohorts before the challenge, early (5–10 min) after the challenge and late (30–35 min) after the challenge. Supplementary Figure S4 also shows side-by-side comparisons between the correlation matrices of both cohorts, to which the *Z*-Scores in Figure 4 correspond.

At slow5 (Figure 4A), we found no alterations in global connectivity induced by the ketamine challenge. Similarly, no differences were found at edge level either before or early after the challenge. However, at the late timepoint, 30–35 min post-challenge, we found a significantly increased coupling between BLA and HPF (*p* < 0.05, FDR-corrected), as well as between the auditory and ectorhinal cortices (*p* < 0.05, FDR-corrected).

In the slow 4 band (Figure 4B), we detected an increase in connectivity early after the challenge in the ketamine cohort (*r* = 0.39 ± 0.04) compared to the saline cohort (*r* = 0.32 ± 0.03), peaking in the block 6–11 min post-challenge. Edge-level alterations were sporadic. Importantly, although the connectivity between HPF and CPu appeared increased at the early timepoint, we also saw a significant difference in the same edge at baseline (*p* < 0.01). The same applies for the connectivity between Au and Peri (*p* < 0.01), also increased in the ketamine cohort before the challenge. No edge-level alterations were observed at the late timepoint.

At slow3 (Figure 4C) we found the most prominent increases in connectivity directly after the ketamine challenge. The dynamic global connectivity increases started approximately 3 min after the challenge and peaked in the 4.5–9.5 min post-challenge interval, at which the FC in the ketamine cohort (*r* = 0.49 ± 0.04) was almost double compared to the saline cohort (*r* = 0.25 ± 0.03). At edge level, with no significant differences between both cohorts at baseline, connections involving several regions were strongly increased at the early timepoint. The regions with the most significantly increased edges included Peri (8 edges), HPF (5 edges), Au (5 edges), Cg (4 edges), Ect (4 edges), CPu (4 edges), and BLA (3 edges). Notably however, the increases in most edges were of a transient nature, since much fewer significant changes were seen at the late timepoint, where only the connections between HPF and BLA, between HPF and Ect, and between ACg and Au remained significantly increased compared to the saline cohort (*p* < 0.05, FDR-corrected). A number of additional edges involving mainly the BLA, HPF, Ect, and Peri were slightly increased, yet did not survive FDR correction at *p* < 0.05.

In the slow2 band (Figure 4D), as opposed to the other frequency bands, we found a linear time-dependent increasing trend in the dynamic FC of both cohorts, while the differences between them were far less pronounced compared to the slow3 band. We found one altered edge between Pal and TAs (*p* < 0.01 pre-FDR) at baseline. At the early timepoint, we detected increased coupling between Ect and Peri, on one side, and the rest of the cortical areas, on the other (e.g., increased FC between Peri and Au, *p* < 0.05, FDR-corrected). The enhanced FC between BLA and CPu (*p* < 0.05, FDR-corrected) appeared consistent, being detected both at the early and the late timepoints, while weaker late increased FC was observed between BLA and HPF as well (*p* < 0.05 before FDR correction).

The slow1-1 and slow1-2 bands (Figures 4E,F) showed remarkably contrasting readouts. The slow1-1 band, in line with most other frequency bands, indicated increased FC of transient nature following ketamine. On edge level, significant differences between both cohorts were only found at the early timepoint and involved ACg (to Ect, CPu, and BLA) and BLA (to ACg, PreL, S1, and HPF). In contrast, in the slow1-2 band we saw a sharp, non-transient decrease in global connectivity in the ketamine cohort (from *r* = 0.22 ± 0.02 at baseline to *r* = 0.14 ± 0.05 at the early timepoint and *r* = 0.10 ± 0.06 at the late timepoint), while the saline FC remained largely constant. At edge level, it translated into stable striatocortical hypoconnectivity, involving M2 (to M1, S1, Au, CPu, and Pal) the M1 (to M2 and Pal), the S1 (to M1, M2, and Pal), as well as between the ACg and PreL. The significant decreases (*p* < 0.05, FDR-corrected) between ACg and PreL, as well as between M2 and M1, S1, Au, and CPu, respectively remained stable across the acquisition. On a general note, the slow1-2 frequency was the only band at which (A) the FC decreased and (B) the alterations were consistent and stable, not returning towards baseline levels until the end of the measurement.

For a two-by-two comparison of the saline and ketamine cohorts, please refer to Supplementary Figure S4.

### Ketamine induces delayed functional connectivity reductions

**Figure 5:**
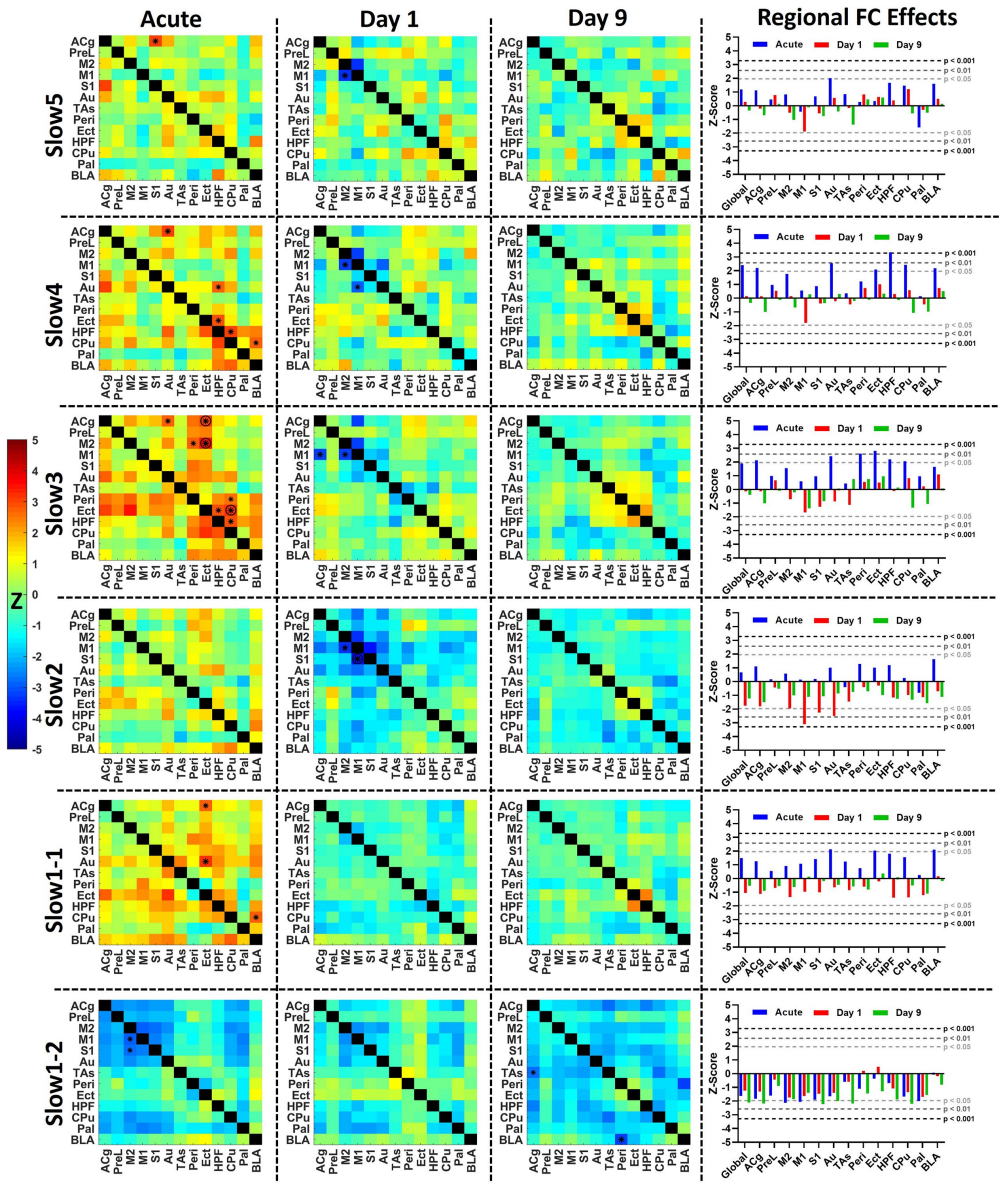
Acute and longitudinal effects of S-ketamine at different FC bands. The matrices indicate the edge-wise *Z*-scores calculated between the readouts of the saline and of the ketamine cohorts at the corresponding timepoints in the respective bands (*p < 0.01, *Z* > 2.58), *O* = *p* < 0.05 (FDR-corrected), combined * and *O* = *p* < 0.01 before FDR correction, *p* < 0.05 after FDR correction. In the right column, the bar diagram indicates the global and regional *Z*-scores summarized for all timepoints. The dotted lines indicate the significance thresholds *p* < 0.05, *Z* > 1.96 (light gray), *p* < 0.01, *Z* > 2.58 (dark gray) and *p* < 0.001, *Z* > 3.29 (black). *Z*, *Z*-score, for the abbreviations of all regions, please refer to Figure 2.

After analyzing the acute effects of ketamine at the different frequency bands dynamically, Figure 5 compares these effects to the longitudinal effects computed at day 1 and day 9. Similarly to Figure 4, the matrices indicate statistical differences between both cohorts at the respective timepoints, for a side-by-side comparison, please refer to Supplementary Figure S5.

In the left column of Figure 5 the average acute effects over the 35 min following the challenge of S-ketamine compared to saline are shown as *Z*-score matrices. The strongest acute effects could be observed, as shown in Figure 4, in the slow 3 band, where FC increases involving Peri, Ect, HPF, M2, and Cg were observed, accompanied by reduced FC in slow1-2 mainly within and between the cortex and the striatal regions.

**Figure 6:**
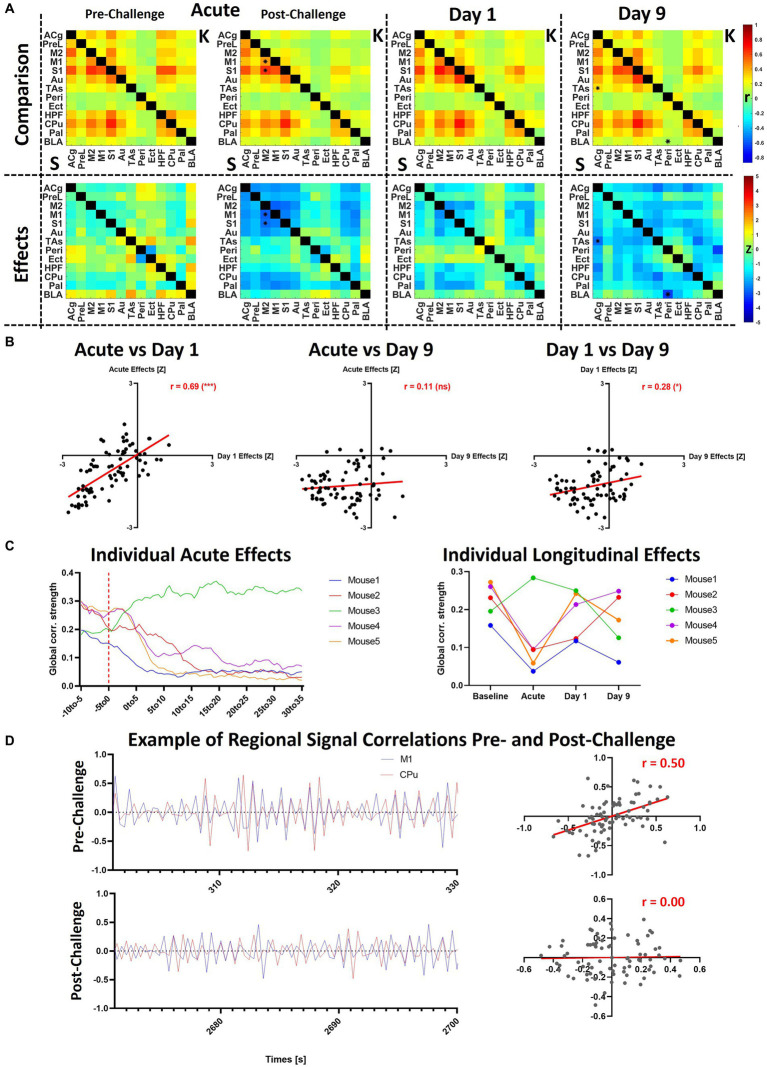
Additional analyses of slow1-2 acute and longitudinal effects. **(A)** The saline and ketamine FC are compared for the acute acquisition at pre- and post-challenge, at day 1 and day 9 (upper row: comparison between the connectivity matrices, saline readout underneath diagonal (indicated using “S”), ketamine cohort above diagonal (indicated using “K”); lower row: *Z*-scores indicating differences between the cohorts). **(B)** Correlations between the *Z*-scores obtained in the acute condition, at day 1 and at day 9. **(C)** Acute and longitudinal global FC effects in the five mice having underwent measurements at all timepoints. **(D)** To exemplify the correlation differences before and after the challenge in a single animal, we plotted the signals in the M1 and CPu in the first 30 s of the pre-challenge period taken as baseline (301–330 s after scan start) and last 30 s of the scan (2671–2,700 s after scan start). The scatter plots indicate the correlations (Pearson’s *r*) between both signals. *p < 0.01, *Z* > 2.58. S, saline; K, ketamine; *r*, Fisher’s *z*-transformed Pearson’s *r* correlation coefficient; *Z*, *Z*-score. For the abbreviations of the regions, please refer to Figure 2.

At day 1, no significant increases were apparent at any band, yet decreased FC between M1 and M2 was seen from slow5 to slow2 (*p* < 0.01 pre-FDR correction), between M1 and S1 at slow2 (*p* < 0.05, FDR-corrected) as well as between M1 and ACg at slow3 and slow2 (*p* < 0.01 pre-FDR correction). At slow2, the regional FC of M1 (*p* < 0.01), S1 (*p* < 0.05), and Au (*p* < 0.05) were significantly decreased (right column). In slow1-2 several edges were decreased at *p* < 0.05 pre-FDR correction involving M2 and Pal (not indicated by asterisks), yet interestingly the pattern of reductions was qualitatively similar to the corresponding acute decreases, albeit less pronounced. This aspect was further investigated in Figure 6.

In contrast, at day 9 no differences could be found between both cohorts from slow5 to slow1-1 at either edge or regional strength level. The only frequency bands where changes were present was slow1-2, including decreases between BLA and Peri (*p* < 0.01) at edge level and reductions (*p* < 0.05) in global (*Z* = −2.1), ACg (*Z* = −2.2), S1 (*Z* = −2.2), Au (*Z* = −2.0), TAs (*Z* = −2.1), and CPu (*Z* = −2.2) FC strengths, while decreases approaching significance levels were observed in a number of other regions.

For a two-by-two comparison of the saline and ketamine cohorts, please refer to Supplementary Figure S5.

### Heterogenous across-subject reductions of FC in the slow1-2 frequency band at all timepoints

As shown in the previous figures, both the acute and longitudinal effects observed in the slow1-2 band were more stable compared to the other bands, although the between-subject variability was higher. To further investigate these aspects, we directly compared the observed changes at the different timepoints and additionally, we analyzed the respective changes occurring in each of the 5 individual mice having underwent all measurements (Figure 6).

Figure 6A summarizes the effects in the slow1-2 band, underlining the initiation of the observed changes directly after the ketamine challenge. The qualitative observation of similarity between the acute changes and those at day 1 is additionally supported (Figure 6B) by the very robust correlation of the respective *Z*-scores (*r* = 0.69, *p* < 0.001). However, at day 9, the observed changes do not present the same topography, not correlating with the acute changes (*r* = 0.11, *p* = 0.27) and only relatively weakly with the effects at day 1 (*r* = 0.27, *p* = 0.05). Figure 6C sheds light onto the observed inter-subject variability following the ketamine challenge. At the acute stage, we observed sharp decreases in the global FC in 4 out of 5 mice, yet remarkably, the connectivity of mouse 3 increased directly after the challenge. Furthermore, at day 1, the FC of all four mice for which it had decreased acutely recovered to differing extents, yet remained under baseline levels (mouse 1: *r* = 0.16 at baseline ➔ *r* = 0.04 acutely post-ketamine ➔ *r* = 0.11 at day 1; mouse 2: *r* = 0.23 at baseline ➔ *r* = 0.09 acutely post-ketamine ➔ *r* = 0.13 at day 1; mouse 4: *r* = 0.26 at baseline ➔ *r* = 0.10 acutely post-ketamine ➔ *r* = 0.21 at day 1; mouse 5: *r* = 0.27 at baseline ➔ *r* = 0.06 acutely post-ketamine ➔ *r* = 0.24 at day 1). Similarly, for mouse 3, for which it had increased acutely after ketamine, global FC remained at a higher level at day 1 (*r* = 0.20 at baseline ➔ *r* = 0.28 acutely post-ketamine ➔ *r* = 0.25 at day 1). However, at least with regards to global FC effects, no consistent pattern of individual changes could be observed at day 9.

To visualize the correlation and lack of correlation between cortical and subcortical regions before and after the challenge, respectively, we plotted the exemplary filtered signals of M1 and CPu over periods of 30 s during the two phases of the scan (Figure 6D). In the presented subject, the correlation between both regions decreased from *r* = 0.5 in the first 30 s of the period defined as baseline to *r* = 0 in the final 30 s of the scan, 35 min after the challenge. In Supplementary Figure S6, raw timecourses, as well as timecourses filtered at all other frequency bands are presented for the same subject and the same two regions. Additionally, the power spectra of the two regions during the challenge scan are shown and compared to the corresponding power spectra of the same subject during the baseline scan.

## Discussion

### Functional integration at different frequency bands

Our study is to our knowledge the first performed with either fMRI or fUS to demonstrate the persistence of FC in mice over different frequency bands. The relative FC strengths detected are very well in line with human studies performed using fMRI, indicating the strongest FC in the slow4 and slow 3 bands and decreased FC at slow2 and the lower range of slow1, defined here as slow1-1, between 0.5 and 0.75 Hz, where FC was consistently lowest across all groups and timepoints (Gohel and Biswal, 2015). While this aspect underlines the potential translatability of frequency-specific FC, our data intriguingly show that at higher frequencies, stronger FC can be derived in slow1 in the band between 0.75 and 1.25 Hz defined here as slow1-2. Importantly, we calculated both repeatability and reliability measures for all studied frequency bands and showed that both the three slower bands, slow5, slow4 and slow3, together comprising the standard range of frequencies at which FC is inferred, as well as the higher bands, slow2, slow1-1 and slow1-2, are similarly reproducible and reliable. In fact, the least reliable group-level readouts were extracted from the slow5 band. A further important observation was, however, that between-subject variability regarding global FC strengths was increased at higher frequency bands, particularly in slow1-2, between 0.75 and 1.25 Hz.

Importantly, differently to all previous papers (Boubela et al., 2013) investigating frequency-specific FC using fMRI, we performed our study using fUS. This aspect is essential, since some of the results on this topic generated with fMRI have been debated due to the use of regression for denoising, shown to potentially induce artificial correlations into the data (Chen et al., 2017). The Power Doppler signal measured from fUS is inherently free of respiration and heart-rate artifacts after the application of the SVD filter (Macé et al., 2011; Deffieux et al., 2018). Here, the only preprocessing steps applied to the Power Doppler signals before calculating the correlations were scrubbing and filtering to the respective frequency bands. Our readouts are therefore not only reliable and reproducible but are also not likely to be influenced by our preprocessing pipeline. It must be however stated that, although no particular unwanted effects induced by the SVD filter early in the pipeline have been reported, these cannot be completely excluded and require further investigation in the future.

We used the slow frequency bands as introduced using electrophysiology by Buzsaki et al. (Buzsaki and Draguhn, 2004), demonstrating concomitant cortical oscillations in the slow bands between 0.01 and 1.5 Hz, the EEG bands between 1.5 and 80 Hz and the fast bands between 80 and 600 Hz (Buzsaki and Draguhn, 2004). Although the hemodynamic signals captured by fMRI and fUS have different physiological bases to the ones recorded by EEG, associations have been made between both readouts, showing that crosstalk between the frequency bands is a distinctive feature of FC (Penttonen and Buzsáki, 2003) and that several processes can coexist at different temporal scales within the same brain regions, underlining the importance of investigating oscillations at different frequency bands (Buzsaki and Draguhn, 2004). However, although previous fMRI studies have indicated that the readouts generated are likely of physiological relevance (Trapp et al., 2018; DeRamus et al., 2021), and task-evoked studies have also observed fast BOLD responses in both humans (Lewis et al., 2016; Frühholz et al., 2020), and animals (Cao et al., 2019), the mechanisms of the fast BOLD signals are still under debate (Chen et al., 2021). Previous work has shown that, for stimulus-invoked BOLD responses, stimulus intensity and technical specifications such as spatial resolution can reveal faster hemodynamic responses compared to the canonical model (Chen et al., 2021). Similarly, using fUS, Aydin et al. demonstrated faster HRF occurring after shorter odor stimulations (Aydin et al., 2020). It could therefore be speculated that spontaneous hemodynamic oscillations, which are inherently of smaller magnitude compared to evoked responses, may be even faster (Chen et al., 2021). Additionally, very good spatial resolution, as our fUS setup provides, could also contribute towards finding faster hemodynamic oscillations, as also discussed in the mentioned publication (Chen et al., 2021).

### Capturing S-ketamine induced functional connectivity effects

While a significant body of literature exists on the effects of ketamine using fMRI, the heterogeneity of the results is as large as that of the study designs, as comprehensively summarized in a recent review on this topic (Kotoula et al., 2021). Several factors appear to impact the readouts, including (1) are the effects acute or delayed, (2) was S-ketamine applied to healthy controls, medicated or unmedicated patients, (3) was S-ketamine or racemic ketamine administered or (4) what was the administration protocol, just to name a few. To our knowledge, no study using fUS to delineate the effects of S-ketamine exists, while the previous reports using fMRI were performed at the standard frequency range between 0.01 and 0.1 Hz.

Furthermore, no literature exists describing the acute effects of S-ketamine in mice. Therefore, to directly compare our data between the slow 5 and slow 3 frequency bands, ranging from 0.01 to 0.198 Hz with previous literature, we focused on studies performed in healthy humans. For all these three bands, we exclusively observed increases directly after S-ketamine injection. The increased FC was most pronounced at slow3 and peaked after approximately 10 min. While the initial increases were not global, they did involve numerous correlations between several regions, such as the hippocampus, caudoputamen and amygdala, but also the anterior cingulate, ectorhinal, secondary motor and auditory cortices. Importantly, most changes were highly transient, many edges returning to baseline levels before the end of the acquisition. Nonetheless, a couple of edges remained increased until the end of the measurement, including the FC between the cingulate and the auditory cortex. Taken together, current evidence mainly indicates hyperconnectivity after acute ketamine administration (Kotoula et al., 2021). Increased frontostriatal connectivity, in line with our findings, has been shown under acute ketamine previously, a feature which correlated moreover with the positive symptoms and dissociative effects of ketamine (Dandash et al., 2015). Other studies have reported increases in FC between the hippocampus and frontal cortical areas including dorsolateral prefrontal cortex (Grimm et al., 2015), precuneus, cingulate and premotor cortex, but also with the basal ganglia (Khalili-Mahani et al., 2015). Niesters et al. also reported ketamine-induced hyperconnectivity between the hippocampus, cingulate, auditory and somatosensory cortex, along with a number of other areas (Niesters et al., 2012). These results were also mirrored in our readout particularly in the slow 3 band. Unfortunately, it is difficult to directly compare the temporal patterns of hyperconnectivity observed in our data with previous literature since the mentioned studies did not assess the changes in connectivity at the same high temporal resolution as we did in the present study using the sliding window approach, only evaluating FC as a static measure. Additionally, in most previous studies ketamine was applied as a bolus followed by a constant infusion, leading to different pharmacokinetics. Our data are however in line with the reported timeline of dissociative effects of ketamine, occurring already within minutes after administration (Krystal et al., 2005) and expand the knowledge of their potential functional underpinnings.

Much fewer studies have reported the effects of ketamine after 24 h or longer in healthy controls. In line with our findings, these studies report, in contrast to the acute effects, mostly decreased FC between the anterior and posterior cingulate cortices, as well as between the anterior cingulate cortex and the prefrontal cortex, the former finding correlating with the magnitude of the psychotomimetic effects (Scheidegger et al., 2012; Lehmann et al., 2016). Intriguingly, in depressed patients, most previous studies indicated increases in FC induced by ketamine at 48 h after administration, although these changes did not persist after 10 days (Evans et al., 2018; Kotoula et al., 2021; Mkrtchian et al., 2021). The reduced FC in our data occurred in the anterior cingulate cortex, but also involved the primary and secondary motor cortices, not reported in previous studies. Since ketamine is known to acutely induce hyperlocomotion (Irifune et al., 1991), which is also in line with the hyperconnectivity seen here in the motor cortex and striatum as well, the hypoconnectivity observed in the same regions at day 1 may represent the corresponding rebound. Furthermore, disrupted connectivity of the sensory and motor networks, observed by us across all frequency bands at day 1, has also been reported in first-episode, drug-naïve schizophrenia patients and predicted the improvement in positive symptoms after medication (Zhang et al., 2019).

### Temporal consistency and between-subject heterogeneity of high-frequency FC effects

Although decreases in connectivity could be seen at day 1 in all frequency bands, no changes were observable at day 9, except in the slow1-2 band. Despite having the highest between-subject variability, the respective subject-level effects, comprising four decreases and one increase following the application of ketamine, were temporally the most stable. Moreover, in terms of directionality, the changes persisted in all five subjects at day 1, albeit being reduced in magnitude. Also, we found a very strong correlation between the patterns of the group-level acute effects of ketamine in slow1-2 and the changes observed at day 1. Importantly, the dynamic FC assessment had already indicated that the changes in this band were most stable temporally during the acute acquisition, the observed hypoconnectivity persisting over the course of the measurements. Therefore, we speculate that this frequency band may be the only one where observed acute changes could be directly linked to more delayed alterations. Notably, in all other frequency bands acute hyperconnectivity was accompanied by delayed hypoconnectivity. At day 9 the global hypoconnectivity in slow1-2 increased in magnitude, however exhibited different patterns, only slightly correlating with the changes at day 1. Also, no consistency could be found at subject level regarding the respective alterations at days 1 and 9, indicating that the effects are not linear and that more frequent measurements and potentially more subjects are required to piece together the temporal evolution between alterations at day 1 and day 9. Regarding the differing between-subject directionality of the acute and day 1 effects, as well as the heterogeneity seen at day 9, it has been reported recently that ketamine effects can be very variable across subjects (Moujaes et al., 2022). Notably, one of the main conclusions of the study above indicates the inter-individual variability as “robust.”

Decreased corticostriatal connectivity has recently been reported as a hallmark of psychosis using dynamic causal modeling, while striatal connectivity was associated with the striatal dopamine synthesis capacity (Sabaroedin et al., 2022). Reduced connectivity between the executive striatum and the anterior cingulate cortex has also been shown in schizophrenic patients previously (Li et al., 2020) and decreased dorsal corticostriatal connectivity has been reported to correlate with positive psychosis-like experiences in healthy individuals (Sabaroedin et al., 2019). At day 9, we found decreased connectivity between the cingulate cortex and the temporal association areas, comparable to reductions in the cingulo-opercular network reported in humans, disturbed salience processing being also proposed as a biomarker of schizophrenia (Miyata, 2019; Culbreth et al., 2021). Less is known on the potential interactions between the amygdala and the perirhinal cortex, even though the two regions are strongly connected (Weston, 2018) and suggested to be involved together in the emotional enhancement of memory (Perugini et al., 2012; Laing and Bashir, 2014). It would be of tremendous interest for future studies to investigate the observed reductions in slow1-2 together with behavioral alterations and identify associations between both readouts.

### Study limitations and strengths

The biggest limitation of our study are the relatively low *n*-numbers. Nonetheless, we found that already at *n*-numbers as low as 5 animals per timepoint reproducible and reliable readouts can be generated at every frequency band. However, the higher between-subject variability in the highest frequency band at both rest and in terms of ketamine effects indicates that a larger cohort may be necessary.

Furthermore, our data was only acquired in one hemisphere to be able to cover a larger number of brain areas. This aspect is due to the limitation of the single probe employed in our fUS system; new technical developments, for instance 2D arrays, enable the acquisition of data from both hemispheres, while covering even more regions on the anterior–posterior axis, such as the midbrain (Rabut et al., 2019). Another currently available alternative to cover more brain areas are motorized probes (Macé et al., 2018; Bertolo et al., 2021; Brunner et al., 2022). However, performing scans using a motorized probe has the inherent trade-off of a poorer temporal resolution. For instance, in our setup, acquiring three slices instead of one would enable us to acquire one frame every 2.4 s, corresponding to a frequency slightly above 0.4 Hz, which, in turn would only enable the analysis of frequency bands up to ~0.2 Hz. Finally, within our 2D slice, the spatial resolution of fUS would have enabled a finer parcellation into several smaller brain areas. In this study we however chose to focus on macrocircuits, rather than microcircuits. This is the reason why we also chose the slightly unconventional and less widely-reported approach of employing an oblique slice (Grohs-Metz et al., 2022), in line with our aim to cover a large variety of different areas, networks and circuits, ranging from the amygdala to the prefrontal cortex.

On a general note, the single-slice acquisition used in fUS is certainly a major disadvantage compared to fMRI, for which whole-brain acquisitions are standard. This is especially the case for pharmacological challenges and drugs expected to exert their effects across the entire brain, such as in S-ketamine in the present study. Developments in technology to close this existing gap to fMRI will benefit pharma-fUS and functional ultrasound in general. Additionally, as mentioned above, literature on the effects of different fMRI preprocessing strategies have highlighted the potential artifacts introduced into the data by certain steps, such as nuisance regression (Chen et al., 2017). Similar studies are required in the future to (a) elucidate the potential side-effects of the SVD filter applied routinely in fUS studies and (b) determine the most appropriate preprocessing strategies in general.

Next, direct vasoactive effects of ketamine, known to increase for instance blood pressure (Liebe et al., 2017), cannot be completely excluded from having an influence on our readouts. For instance, anesthetics changing blood pressure have been reported to potentially affect Mayer waves (Shin et al., 2011), which lie in frequency ranges coinciding with the Slow3 and Slow2 bands. However, in addition to us using subanesthetic doses of ketamine, we speculate that coherent vasomotor activity may rather have a positive impact on the interregional correlations of hemodynamic signals. Therefore, we would expect that disturbing such waves would rather lead to a decrease in correlations, while in Slow3 and Slow2 we report increases after acute ketamine administration. Nonetheless, it has been shown that vasoactive substances may strongly affect neurovascular coupling (Ionescu et al., 2023), therefore their neuronal effects and their interpretation should always be treated with caution when employing hemodynamic signals. The physiological data we acquired (Supplementary Figure S1) showed largely stable physiology for both cohorts and all four timepoints. We report normal blood oxygenation levels (>90%) for both cohorts and all measurements. Importantly, while we did see increases in heart rate after the ketamine challenge, in accordance previous literature (Liebe et al., 2017), blood oxygenation did not show any alterations. We do not expect changes in heart rate to have a major influence on our signal, since cardiac pulsatility effects are reliably filtered out by the SVD filter (Demené et al., 2015) and because the cardiac pulse has a much higher frequency than the bands we used to filter our signals (~230 bpm before the challenge increasing to ~250 bpm after the challenge correspond to 3.8 and 4.2 Hz, respectively). We did see a gradual increase in heart rate, also previously reported in rodents (Sirmpilatze et al., 2019), however it occurred for both cohorts and at all timepoints.

Finally, our study was performed in sedated mice. Although the level of medetomidine sedation was kept as low as possible, potential effects on neuronal activity, hemodynamics or direct interactions with the applied challenges cannot be excluded (Jonckers et al., 2014, 2015). Recently, Ferrier et al. showed that for medetomidine the dosage is particularly important for the level of sedation and for the FC readout (Ferrier et al., 2020). The dose used in the present work nearer to the light sedation described in the publication by Ferrier et al., shown to preserve to a large extent the awake functional connectome. Additionally, the reliability of the FC between timepoints in the saline cohort indicates that longitudinal effects of repeated sedation were largely negligible. In general, however, the capability of fUS to enable awake animal imaging (Brunner et al., 2020; Ferrier et al., 2020) opens new avenues for future studies and could potentially offer a cleaner picture of pharmacological effects, without the confounds of sedation.

The main strengths of our studies were the use of fUS and the comparison of acute and longitudinal effects in the same cohorts. The current gold standard BOLD-fMRI suffers from the convoluted nature of its signal and can be prone to artifacts, especially when filtered at high frequencies, as discussed above. With its higher sensitivity and spatiotemporal resolution, fUS is ideally placed to investigate drug effects, although pharmaco-fUS studies are still sparse up to date (Rabut et al., 2020; Vidal et al., 2020, 2022).

## Conclusion

Our study pioneers the evaluation of FC slow frequencies beyond the standard ranges of 0.01–0.2 Hz using fUS, demonstrating the robustness and reliability of acquired readouts. By using this analysis, combined with the mentioned superior technical capablilities of fUS, similarly designed studies may strongly contribute to the investigation of acute and delayed effects of pharmacological interventions.

## Data availability statement

The raw data supporting the conclusions of this article will be made available by the authors, without undue reservation.

## Ethics statement

The animal study was reviewed and approved by Regierungspräsidium Tübingen.

## Author contributions

GG-M performed all experiments. TI performed the analysis, conceptualized, and wrote the article. BH supervised the project. GG-M and BH revised the manuscript. All authors contributed to the article and approved the submitted version.

## Funding

The study was funded in its entirety by Boehringer Ingelheim Pharma GmbH & Co.

## Conflict of interest

TI, GG-M, and BH are employed by Boehringer Ingelheim Pharma GmbH & Co.

## Publisher’s note

All claims expressed in this article are solely those of the authors and do not necessarily represent those of their affiliated organizations, or those of the publisher, the editors and the reviewers. Any product that may be evaluated in this article, or claim that may be made by its manufacturer, is not guaranteed or endorsed by the publisher.
