# Supplementary material for: Functional ultrasound detects frequency-specific acute and delayed S-ketamine effects in the healthy mouse brain
Source: Front Neurosci. 2023 May 17;17:1177428. doi: 10.3389/fnins.2023.1177428 (PMC10229773; doi:10.3389/fnins.2023.1177428)
Supplement: Supplementary file 1 [file Data_Sheet_1.docx]

# Supplementary Figures


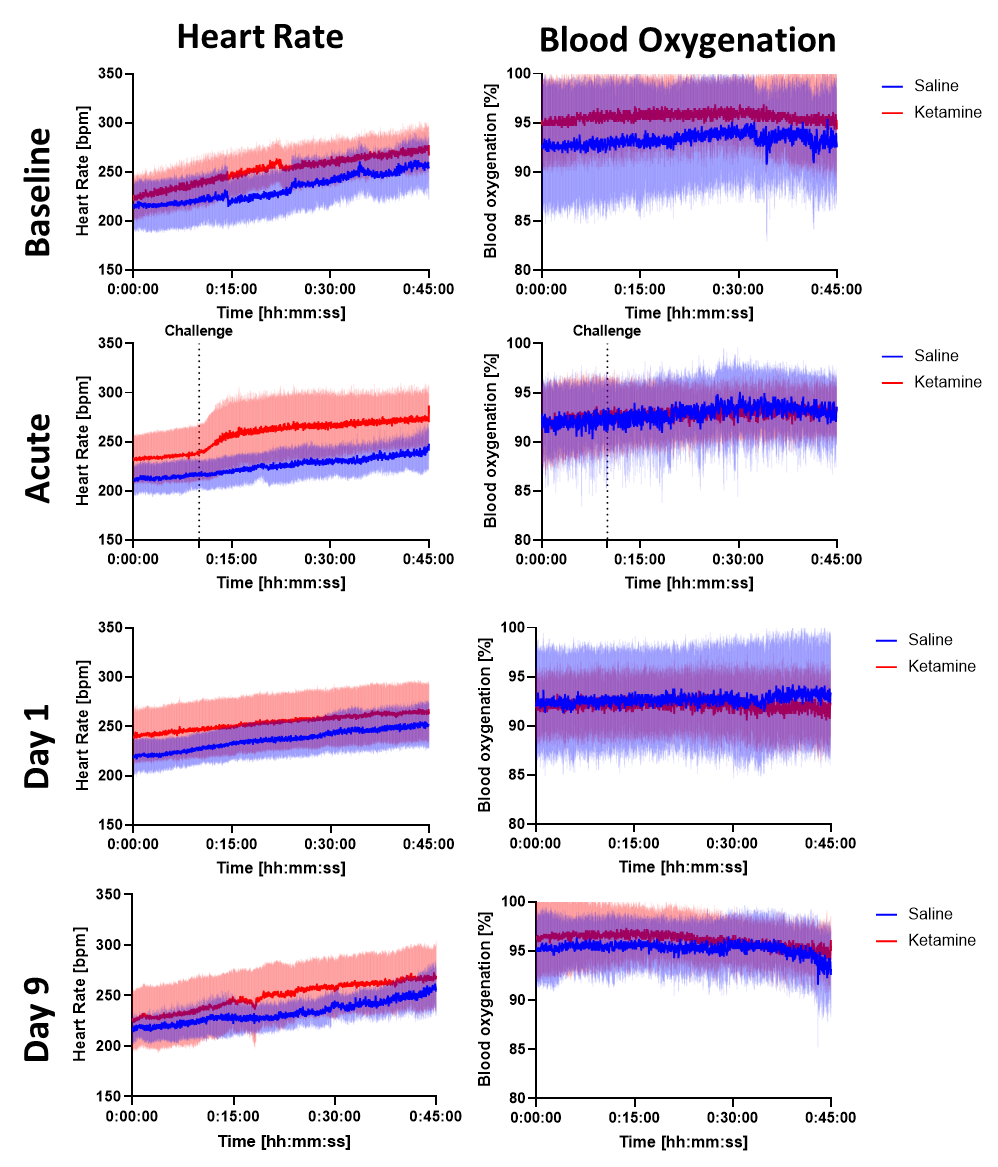


**Supplementary Figure 1: Heart rate and blood oxygenation for both cohorts and every measurement.** Data are presented as mean ± SD, for the acute measurements dotted lines indicate the moment of the challenge


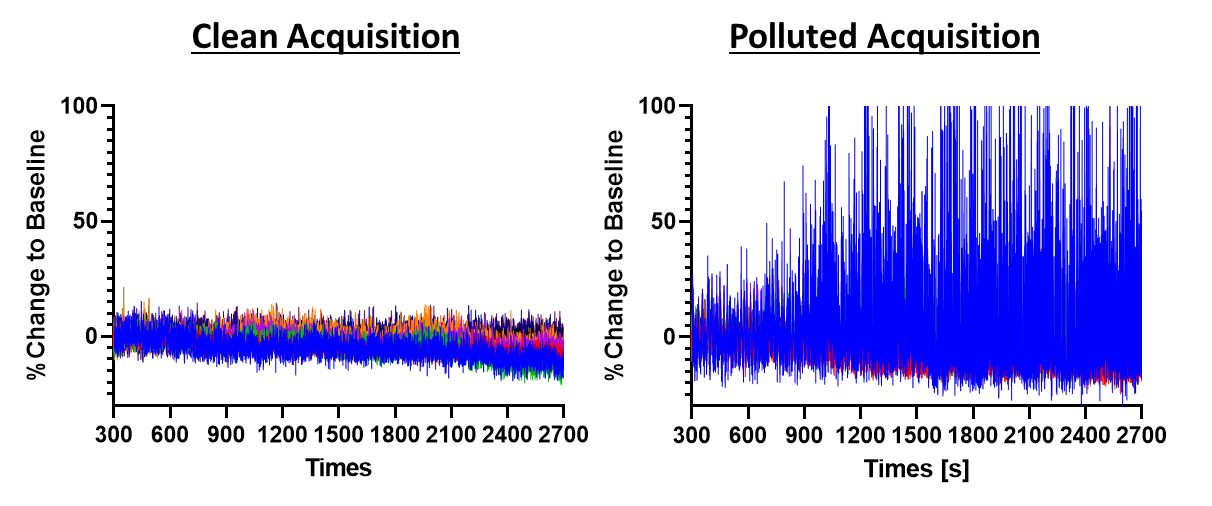


**Supplementary Figure 2: Examples of a clean acquisition and of a polluted acquisitions excluded from the scan.** Each line indicates Power doppler signals relative to baseline (5-10 minutes after scan start) for each region between 5 minutes after the scan start and the end of the scan.


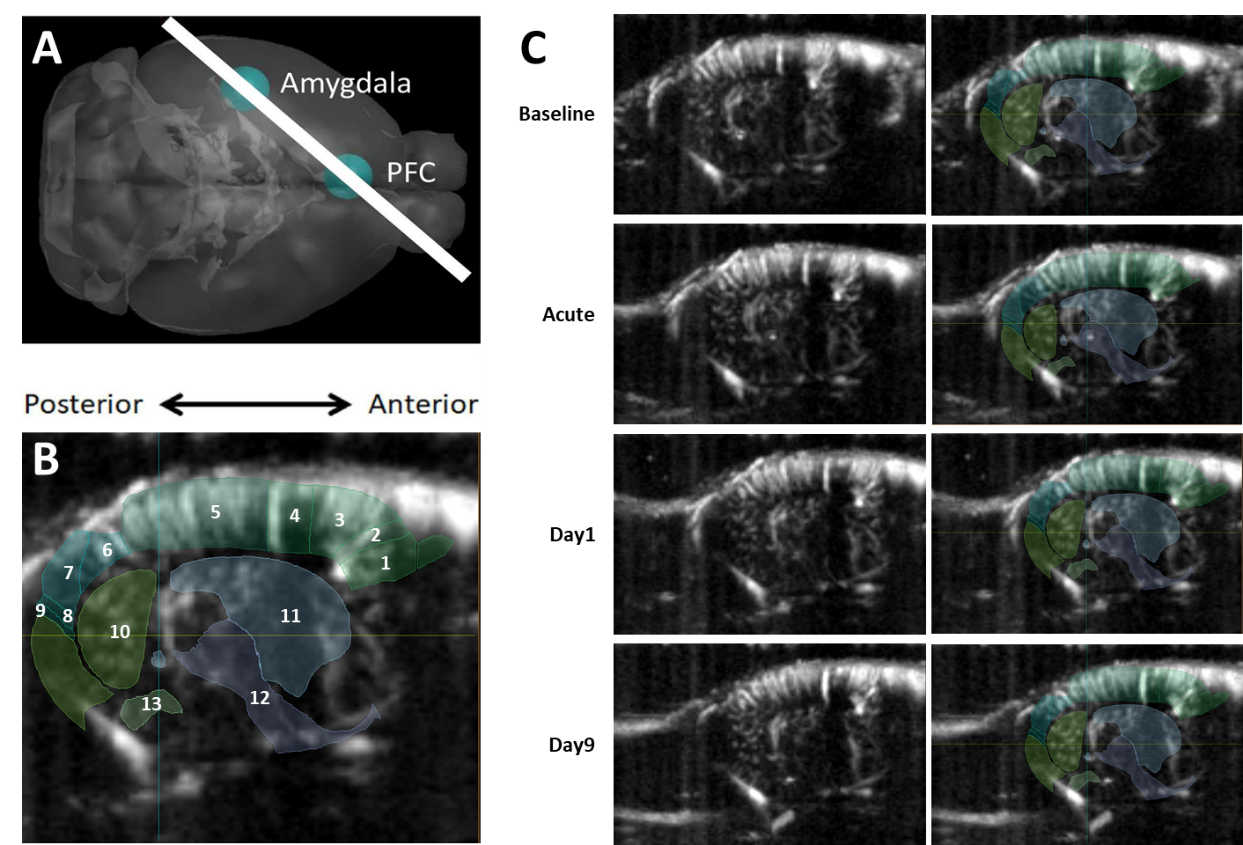


**Supplementary Figure 3: (A)** Illustration of probe position (figure reproduced, with permission, from ^38^), **(B)** ROIs used for signal extraction: 1 – medial prefrontal cortex, 2 – anterior cingulate cortex, 3 – secondary motor cortex, 4 – primary motor cortex, 5 – somatosensory cortex, 6 – auditory cortex, 7 – temporal association cortex, 8 – ectorhinal cortex, 9 – perirhinal cortex, 10 – hippocampal formation, 11 – caudoputamen, 12 – globus pallidum, 13 – basolateral amygdala. **(C)** Exemplary Power Doppler images with and without overlaid ROIs across the four different sessions from one subject.


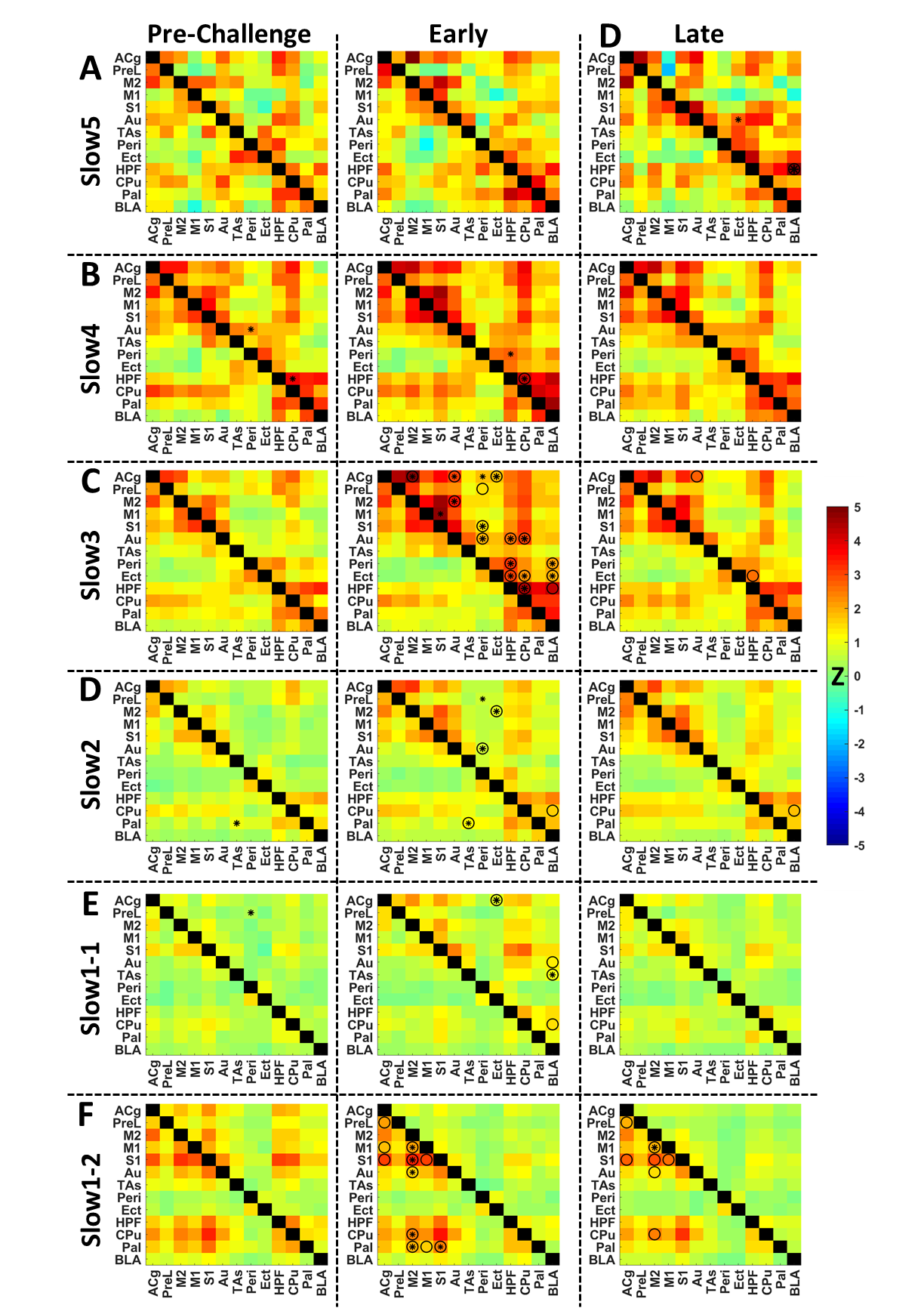


**Supplementary Figure 4: Two-by-two comparison matrices (saline cohort below diagonal, ketamine cohort above diagonal) corresponding to the acute effects shown in Figure 4.**


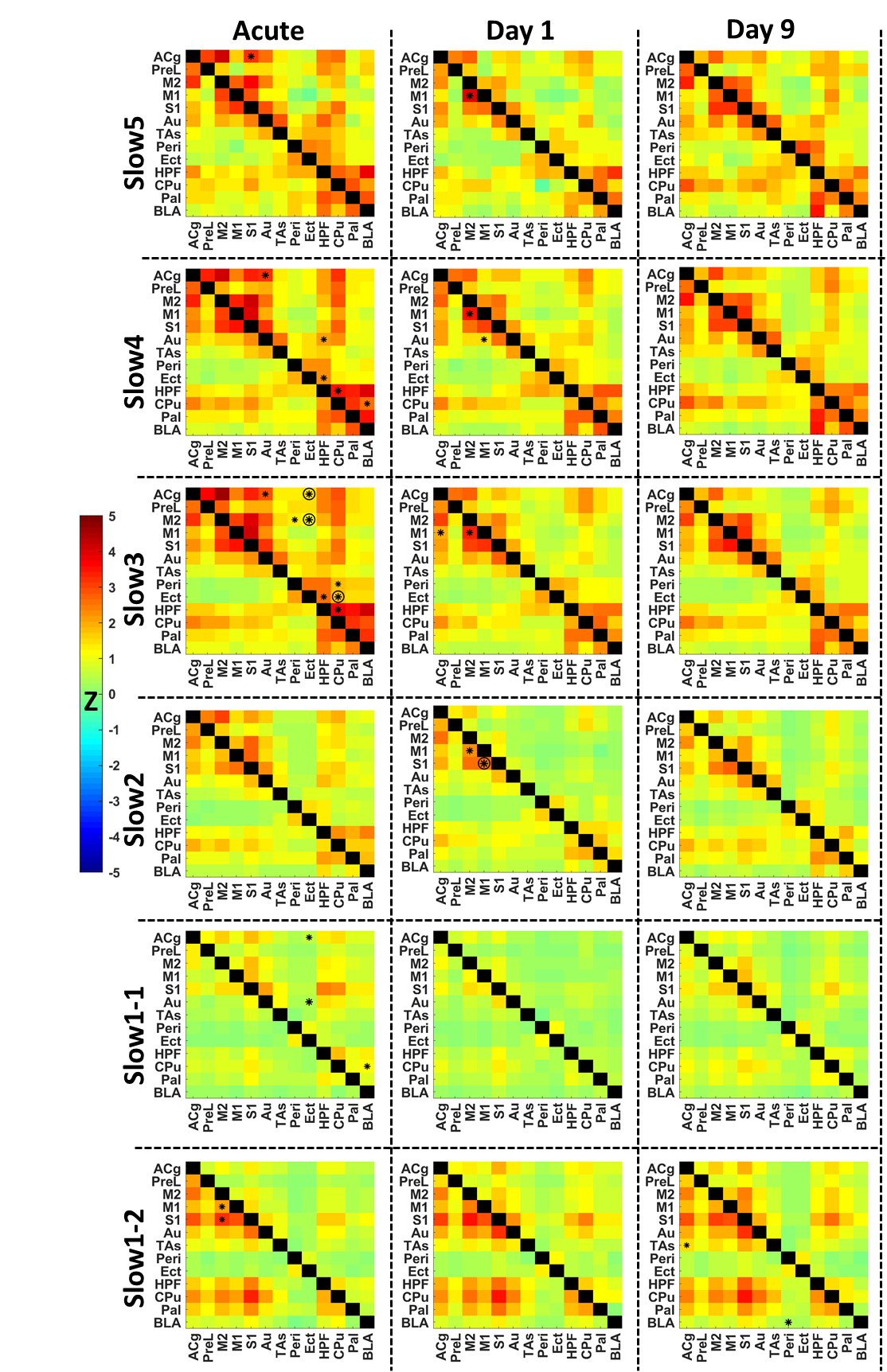


**Supplementary Figure 5: Two-by-two comparison matrices (saline cohort below diagonal, ketamine cohort above diagonal) corresponding to the longitudinal effects shown in Figure 5.**


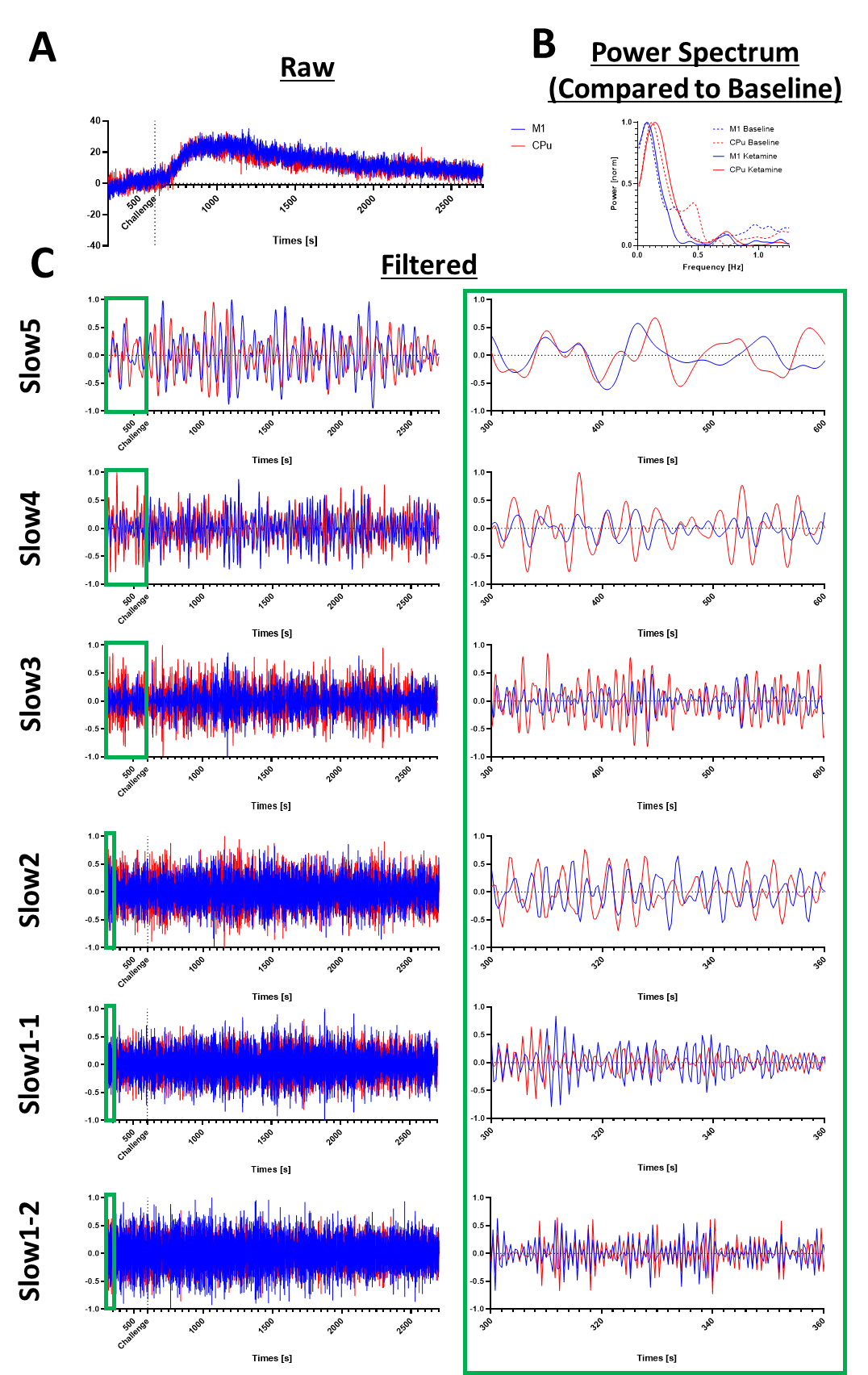


**Supplementary Figure 6: Depiction of raw and filtered timecourses of M1 and CPu for a single subject’s ketamine challenge scan. (A)** Raw timecourses showing increases directly after ketamine administration, **(B)** Normalized power spectra of both regions, compared to the power spectra of both areas in the same subject during the baseline scan, showing decreases in the frequency range corresponding to the slow1-2 band. **(C)** The left graphs show filtered signals for all bands over the entire course of the scan. The green boxes indicate shorter periods (5 minutes for slow5, slow 4 and slow3, 1 minute for slow2, slow 1-1 and slow1-2) selected for better visualization of the respective signals (shown correspondingly in the right graphs).
